# Structure–Activity Prediction of ACE Inhibitory/Bitter Dipeptides—A Chemometric Approach Based on Stepwise Regression

**DOI:** 10.3390/molecules24050950

**Published:** 2019-03-08

**Authors:** Monika Hrynkiewicz, Anna Iwaniak, Justyna Bucholska, Piotr Minkiewicz, Małgorzata Darewicz

**Affiliations:** Faculty of Food Science, Chair of Food Biochemistry, University of Warmia and Mazury in Olsztyn, Pl. Cieszyński 1, 10-726 Olsztyn-Kortowo, Poland; monika.protasiewicz@uwm.edu.pl (M.H.); justyna.bucholska@uwm.edu.pl (J.B.); minkiew@uwm.edu.pl (P.M.); darewicz@uwm.edu.pl (M.D.)

**Keywords:** ACE inhibitors, bitter dipeptides, backward/forward regression, BIOPEP-UWM database

## Abstract

Forward and backward stepwise regression (FR and BR, respectively) was applied for the structure–bioactivity prediction of angiotensin converting enzyme (ACE)-inhibitory/bitter-tasting dipeptides. The datasets used in this study consisted of 28 sequences and numerical variables reflecting dipeptides’ physicochemical nature. The data were acquired from the BIOPEP-UWM, Biological Magnetic Resonance Databank, ProtScale, and AAindex databases. The calculations were computed using STATISTICA^®^13.1. FR/BR models differed in R^2^ (0.91/0.76, respectively). The impact of C-_at_C(−) and N-Molw(+) on the dual function of dipeptides was observed. Positive (+) and negative (−) correlations with log IC_50_ are presented in parens. Moreover, C-Bur(+), N-_at_H(+), and N-Pol(−) were also found to be important in the FR model. The additional statistical significance of N-bul(−), N-Bur(−), and N-Hdr(+) was reported in the BR model. These attributes reflected the composition of the dipeptides. We report that the “ideal” bitter ACE inhibitor should be composed of P, Y, F (C-end) and G, V, I, L (N-end). Functions: log R_caf._ = *f* (observed log IC_50_) and log R_caf._ = *f* (predicted log IC_50_) revealed no direct relationships between ACE inhibition and the bitterness of the dipeptides. It probably resulted from some structural discrepancies between the ACE inhibitory/bitter peptides and/or the measure of activity describing one of the two bioactivities. Our protocol can be applicable for the structure–bioactivity prediction of other bioactivities peptides.

## 1. Introduction

It is well documented in the literature that peptides derived from food proteins exhibit diversity of bioactivities, such as antihypertensive, antioxidative, antithrombotic, antidiabetic, immunomodulating functions, etc. [1]. Peptides’ ability to inhibit angiotensin converting enzyme (ACE; EC 3.4.15.1) has been the most extensively described such bioactivity in papers compared with other biological functions [2]. Moreover, ACE-inhibiting peptides have been identified in proteins originating in practically all food sources [3].

Generally, ACE is involved in blood pressure regulation. Briefly, it transforms angiotensin I, a decapeptide with the sequence DRVYIHPFHL, into angiotensin II (an octapeptide: DRVYIHPF) and C-terminal dipeptide histidyl-leucine (HL). The transformation of angiotensin I into angiotensin II leads to vasoconstriction and finally to elevated blood pressure. In turn, ACE catalyzes the cleavage of vasodilating bradykinin (RPPGFSPFR). The products of this reaction are as follows: C-terminal dipeptide FR (phenyl-arginine) and RPPGFSP (bradykinin 1–7). Thus, the action of the such inhibiting peptides (e.g., derived from food proteins) affects blood pressure reduction [4,5].

Compared with the synthetic antihypertensive drugs, peptides acting as ACE inhibitors are considered to be milder, non-toxic, and safer [6]. Therefore, some ACE-inhibiting peptides have the potential to be used as diet-originating therapeutic agents in hypertension treatment [7]. This is consistent with the approach of [8] in considering that peptides—as functional food ingredients and nutraceuticals—may help avoid various undesirable side effects associated with organically synthesized chemical medicines and can reduce the costs of drug therapy. Moreover, many therapeutic peptides and proteins are classified as biodrugs and are studied by biotechnologists who seek the production of needle-free and more user-friendly drugs [9]. 

Apart from their biological functions, peptides may affect all five taste sensations, i.e., bitter, salty, sour, sweet, and umami [10,11]. Bitterness is the most common taste that is associated with the peptides generated during food protein hydrolysis. Thus, the formation of off-flavor bitter peptides during proteolysis is considered to be one of the limitations in the production of biologically, chemically, and functionally valuable food hydrolysates, which may negatively affect their practical application in the food and nutrition industries [12].

Although food-originating peptide ACE inhibitors have a beneficial impact on health, they are often carriers of unwanted bitter tastes, which can be an obstacle when considering the enhancement of foods with the abovementioned bioactivity [13]. Thus, scientists study biopeptides, including ACE inhibitors, using in silico, in vitro, and ex vivo/in vivo methods [14,15]. The first approach involves computer modeling (bio- and cheminformatic analyses), including the structure–function prediction of ACE inhibitors [16,17], the second includes, e.g., the identification of peptides in novel food sources and/or optimal technologies of their production [14], and the third one includes studies with humans and animals, in particular, spontaneously hypertensive rats (SHR) [18]. 

In silico methods have been found useful in predicting the properties that determine the bioactivity of peptides resulting from their chemical nature [19]. Such an approach is called quantitative structure–activity relationship (QSAR) and is applied to study “separately” ACE-inhibiting [17] and/or bitter peptides [20]. Our quick search using the words “bitter ACE inhibitors” as a query revealed only a few original papers concerning this subject. Three of them were found in the National Center for Biotechnology Information (NCBI) [21], and two were listed in Web of Science (WoS) [22]. Progress has been observed in computer technologies since the release of these papers. It has contributed to the increasing number of databases providing information about the peptides, programs for providing data on their physicochemical properties, and programs analyzing created datasets using chemometric methods, such as artificial neural networks (ANN), principal component analysis (PCA), multiple regression (MLR) techniques, etc. [19]. Such a variety of tools are suitable to create data matrices, which help to understand the impact of the chemical nature of peptides on their bioactivity/function [20].

Thus, the aim of our study was to predict which specific properties of amino acids forming dipeptide sequences determine their dual function (i.e., ACE inhibition and bitter taste) using MLR variants (i.e., forward and backward stepwise regression).

## 2. Results

Two multilinear regression (MLR) models, namely forward regression (FR) and backward regression (BR), were carried out for two datasets composed of 28 peptidic bitter ACE inhibitors and 20 variables each. An F-test made for the analysis of significance of the estimated models led to rejecting the H_null_ (briefly, independent variables do not affect the dependent variable, meaning log IC_50_). The reason for this was *p* < 0.01 and the relatively high values of the F-test. According to [23], the constructed regression model is considered to be suitable when an F-test or ANOVA analysis is carried out at least at *p* < 0.05. In the FR and BR models, an F-test is often applied as the default criterion to stop the stepwise regression procedure. Three values of α are applied when performing an F-test, namely 0.01, 0.02, and 0.05. However, some authors suggest using only the first two α-values rather than 0.05 to avoid the overfitting of the models [23]. Thus, in our study we applied the α-value of 0.01 to analyze the results of the F-test (see Section 4). The standard estimation error, being an indicator of differences between the observed and predicted log IC_50_, was 0.41 (FR model) and 0.58 (BR model) (Table 1). FR and BR models explained the variance (R^2^) in 91.0 and 76.0%, respectively. Normality Shapiro–Wilk’s (SW-W) test calculated for both regression models led to obtaining *p* > α (α = 0.01). The p-value was 0.254 (SW-W = 0.954) for FR and 0.136 for BR (SW-W = 0.943) (data not shown). 

**Figure 1:**
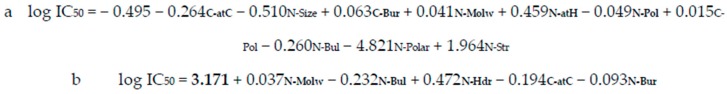
FR (**a**) and BR (**b**) equations explaining the structure–function of the ACE-inhibiting/bitter dipeptides (statistically significant parameters are indicated in bold).

Based on the results shown in Table 1, two equations explaining the impact of the individual variables on the log IC_50_ (Figure 1a,b) were generated. The first equation (Figure 1a) referred to the FR model, which was obtained in 10 steps, while the second one (Figure 1b), concerning the BR model, was calculated in 15 steps. 

A total of five variables were statistically significant in both models (see also Table 1). Additionally, in the case of the BR model, the intercept was also found to be statistically significant. The importance of an individual variable in the bioactivity explanation was represented by a positive and/or negative value of a regression coefficient [17]. Thus, considering log IC_50_ parameter interpretation (the lower its value, the greater the bioactivity of a peptide), negative values of regression coefficients indicate that the higher value of a variable results in the lower final value of log IC_50_ [24]. For example, when looking at Figure 1a, if the molecular weight of the N-terminal residue raises, log IC_50_ will increase. To conclude, to get lower values of log IC_50_, it is better for peptidic bitter ACE inhibitors to be composed of N-terminal low molecular weight amino acids such as G (Molw = 75.07 Da).

The variables negatively correlated with log IC_50_ were as follows: C-_at_C, N-Size, N-Pol, and N-Polar (FR) and N-Bul, C-_at_C, and N-Bur (BR). The positively correlated ones were as follows: C-Bur, N-Molw, N-_at_H, C-Pol, and N-Str (FR) and N-Molw and N-Hdr (BR). 

The ACE-inhibitory activity of the sequences derived from the BIOPEP-UWM database was expressed as IC_50_ [μM] [13] (see Section 4), whereas the bitter taste was defined as R_caf._, namely the bitterness of the peptide related to that of 1 mM caffeine solution [20]. Although our dipeptide data represented sequences with dual function expressed with two different parameters (IC_50_ and R_caf_.), the FR and BR equations were calculated taking into consideration log IC_50_ as a dependent variable. Then, the next step was to compare the dipeptide sequences with approximate observed versus predicted values of log IC_50_, with R_caf._ representing their potential as bitter tastants (see Table 2). 

**Figure 2:**
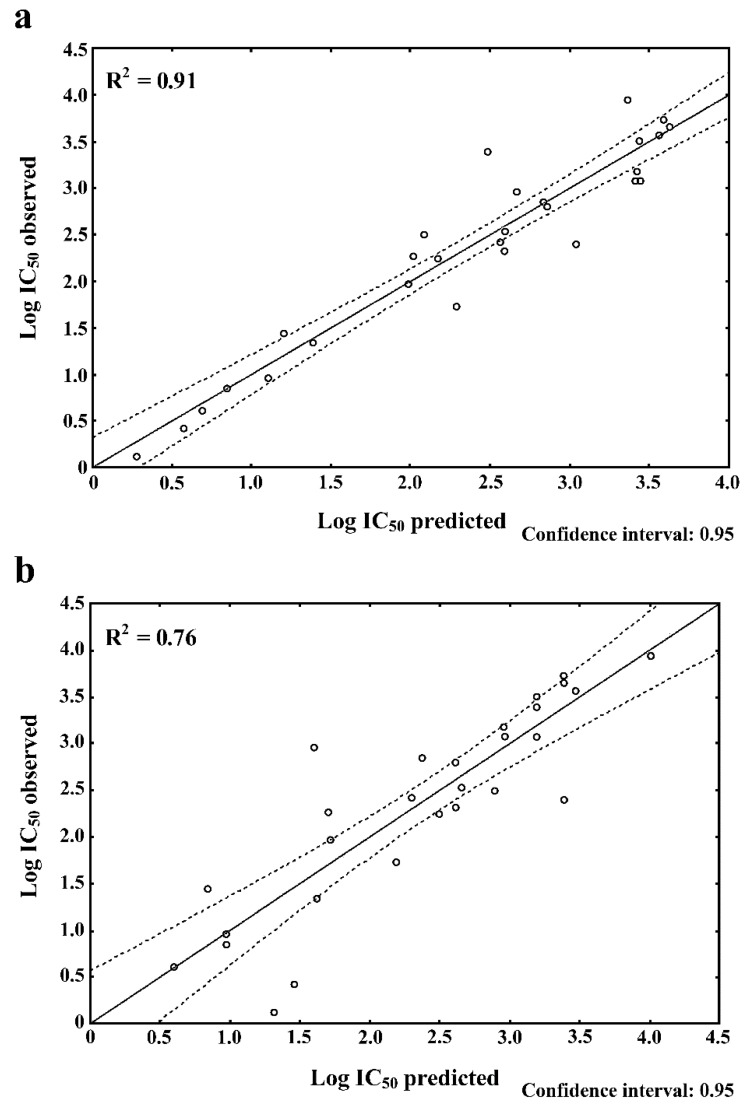
Observed and predicted log IC_50_ of ACE-inhibiting/bitter dipeptides: (**a**) FR and (**b**) BR. The dashed line denotes the confidence interval.

To achieve such data, based on the FR and BR, two linear regression curves were plotted (Figure 2a,b, respectively). They illustrate the distribution of the samples (dipeptides) in the area of a regression curve (solid line) or a confidence interval set by default at 95% (dashed line) considering the observed and predicted values of log IC_50_. The dipeptides possessing approximate observed vs. predicted log IC_50_ values were compared with R_caf_. estimating peptide bitterness. The FR results revealed 15 dipeptides with approximate observed versus predicted log IC_50_ values, whereas BR revealed 14 dipeptides (see Table 2). At total of nine common sequences possessing similar observed and predicted log IC_50_ values were observed in both models. They were as follows: PR, VY, VF, RF, RR, LF, GF, FG, and GV. Moreover, our dataset (see Section 4) was composed of 6 ACE-inhibiting sequences containing P, and four of them (KP, RP, YP, and PR) (Table 2; FR results) possessed approximate observed and predicted values of log IC_50_ values ranging from 0.61/0.69 (PR) to 2.86/2.83 (YP), respectively. Comparing their log IC_50_ with the log IC_50_ of other dipeptides, these values were observed to be relatively low, which indicated a high potential of P-containing dipeptides as ACE inhibitors. When looking at R_caf_. of the P-containing dipeptides (the higher the value, the more bitter the peptide) [20], they ranged from 0.05 (YP) to 1.25 (RP).

Our study results show that the presence of P may determine the ACE-inhibitory activity and bitterness of a dipeptide. This may also be observed when searching databases of peptides, such as the BIOPEP-UWM database of sensory peptides and amino acids, in which the majority of bitter-tasting sequences with different chain length possess P and match the sequences with confirmed ACE-inhibitory bioactivity found in the BIOPEP-UWM database of bioactive peptide sequences [10,25,26].

So far, our abovementioned observations regarding the prediction of the dipeptide structure–ACE-inhibition/bitterness have concerned the association of the particular variables with the log IC_50_. No statistically significant results were found in both models (FR and BR) when directly comparing the ACE inhibition (predicted and observed log IC_50_) and the bitterness (log R_caf_.) of the dipeptides analyzed. The correlations between the abovementioned functions of dipeptides were relatively low (see Table 3). 

The relatively low correlations were obtained in our studies (i.e., R^2^ = 0.32) between ACE inhibition and bitter taste of di- and tripeptides were higher than those obtained by other authors [12]. According to [12], this resulted from the discrepancies between the detailed structural requirements of peptides to act as ACE inhibitors (e.g., the presence of C-terminal bulky aromatic residue and positively charged amino acid in adjacent positions), as well as bitter tastants (e.g., the presence of hydrophobic side-chain C-terminal residue with aromatic amino acid in adjacent positions) [12]. 

## 3. Discussion

According to [13], the structure–bioactivity prediction of peptides showing more than one bioactivity is often difficult, because their biological function is measured for one effect. Thus, by using the BIOPEP-UWM database, a collection of bioactive [26] and sensory peptides [25], our strategy was to find the dipeptide sequences affecting both ACE inhibition and bitterness. 

The main challenge for data analysts and scientists dealing with multivariate analyses, including multilinear regression, is to define which variables can be taken for model construction. Stepwise regression is one of the methods that allows the selecting of variables. Two stepwise regression methods are commonly used, namely forward and backward regression (FR and BR, respectively). Both of them enable building models to find the optimal solutions to a problem [27], such as predicting the impact of independent variables (e.g., numerically expressed properties) on a dependent variable (e.g., the measure of bioactivity) by describing the function of a sample (e.g., peptide) [19]. 

FR and BR are sequential models used to create a final model, which is built by adding or eliminating a predictor (a variable) [28]. Sequentiality relies on building successive models upon a prior model (unless the first model is a final one) when no more predictors based on specific criteria are included/excluded. Briefly, FR starts with no variables in the model. If the p-value of an individual variable is below the α-value, the one with the lowest value is included in the model, remaining in it forever (i.e., it affects the dependent variable). The procedure is continued until no variable reaches the entrance criteria. In BR, no predictors are found when starting. The predictors possessing the highest p-values among the pre-specified level are eliminated with no possibility of returning because their impact on the dependent variable is negligible. The resultant model is reduced and the procedure continues until the analysis of p-values for all remaining variables reaches the entrance criteria [29]. To conclude, the stepwise linear regression-driven approach enables determining the most influencing variables and including the most significant ones in the final analysis [30] and hence was taken into consideration when constructing the models to determine the impact of specific variables on the activity of dipeptides acting as ACE inhibitors and bitter tastants.

Our FR and BR models explained the variance (R^2^) in 91.0 and 76.0%, respectively (see Table 1). The latter value was comparable to the results reported by [31] concerning the application of partial least squares (PLS) in the analysis of structure–ACE-inhibition relations of 58 dipeptide sequences. In turn, our R^2^ = 0.91 approximated the PLS results of structure–bitterness studies of 48 dipeptides, in which R^2^ was 0.93 [31]. Our data confirmed a hypothesis about the normality of distribution [28], which indicated the suitability of FR and BR models for predicting the impact of the variables used for log IC_50_ prediction.

To summarize the statistical facts, although both models applied to the structural characteristics of dipeptide bitter ACE inhibitors possessed relatively low values of standard error of estimation and revealed the impact of five variables that were statistically significant, FR can be theoretically considered to be better than BR. The reason for such a conclusion is the higher values of R^2^ and adjusted R^2^ in the FR model compared with those of the BR model, meaning the model better fit to the data [28]. Thus, we decided to continue the procedure by determining the FR and BR equations describing the structure–activity relationships of the ACE-inhibiting/bitter-tasting dipeptides. As it was said above, the FR model was achieved in 10 steps, while the BR model was achieved in 15 steps. In both models, dependent variables illustrating the bioactivity of the dipeptides was represented by their log IC_50_ being the measure of ACE inhibition. The decision concerning the selection of such bioactivity measure resulted from an overview of scientific reports, according to which the QSAR models for ACE inhibitors were constructed with log IC_50_ [17]. Moreover, logarithms of IC_50_ (precisely, −log IC_50_) collected from the literature were applied for QSAR modeling of 48 bitter dipeptides to enable more efficient validation of new descriptors [32]. To conclude, we used log IC_50_ values as a “more leading” measure of the bioactivity of the analyzed peptides.

Both models (i.e., FR and BR) revealed five variables that were statistically significant in explaining the structure–bioactivity relationship of the dipeptides analyzed (see Table 1 and Figure 1a,b). The comparison of the two equations revealed the existence of two common, statistically significant variables influencing the double function of the dipeptides, namely C-_at_C (negative correlations) and N-Molw (positive correlations). Some common structural features of the ACE-inhibiting peptides that can be associated with bitterness were reported by other authors in their quantitative structural descriptions [33]. According to [33], the bitterness of the peptides was associated with relatively low values of a Balaban index related to the number of atoms in a molecule (the more atoms in a molecule, the lower the Balaban index). This observation matches the impact of C-Molw and C-_at_C on the dual bioactivity of the analyzed dipeptides. This may result from the presence of relatively high molecular weight residues, such as F and/or Y in a dipeptide sequence. According to [34], peptide bitterness was also determined by the presence of the abovementioned amino acids. Moreover, bitterness may be intensified by the presence of G [34], which was found to be statistically significant in our regression equations represented by positive correlations with log IC_50_ of N-Molw (FR and BR models) and N-atH (BR model). Finally, our results concerning the interpretation of variables in the context of their association with the specific amino acids present in bitter ACE-inhibiting dipeptides are also consistent with studies by [35] who found a correlation between the ACE inhibition and bitterness (R^2^ = 0.87) of enzymatic hydrolysates produced from shrimp (*Pandalopsis dispar*) protein byproducts. They observed that ACE inhibition and bitter-tasting hydrolysates were related to the presence of Y, F, L, I, V, and K [35]. 

It is well known that the N- and C-terminal locations of a residue in a sequence determines peptide bioactivity [36]. According to the scientific reports, the presence of the N-terminal G, I, L, and V in a peptide chain is preferable for ACE inhibition, while P, Y, and W are the favored C-terminal amino acids [37,38]. Such observations are consistent with the results obtained in our study. According to the regression models (Figure 1a,b), our potent ACE-inhibiting bitter peptides should be composed of C-terminal amino acids with a relatively high molecular weight (C-Molw), which is affected by an increasing number of carbon atoms (C-_at_C), as well as polarity (C-Pol). This regularity was represented by the presence of C-terminal Y, R, and K of a dipeptide dataset. According to [36], the enhancement of the ACE-inhibitory potential of peptides composed of 2–6 amino acids depended on the increased side-chain hydrophobicity of C-terminal amino acid and the decreased side-chain size of a residue in an adjacent position. It may refer to dipeptides possessing C-terminal F, Y, and/or P. Taking into consideration the N-terminal residues of the dipeptides analyzed, they were composed of amino acids with a relatively low molecular weight (G, I, L) and rather hydrophobic side chains, which was reflected by the positive correlations in our stepwise regression models. According to [17], highly potent dipeptides with an ACE-inhibiting effect should generally be composed of bulky and hydrophobic side chain residues. Hydrophobicity and the total length of a peptide were also indicated by other authors [39] as important parameters affecting the bitterness of single amino acids, as well as di- and tripeptides. 

When looking at both equations, it can be observed that the buriability of the N- and C-terminal residue (N-Bur and C-Bur, respectively) had a statistically significant impact on log IC_50_. This parameter explains how the burial of hydrophobic or hydrophilic amino acids inside a protein sequence contributes to its stability. It is suggested that hydrophilic residues have a negative or minor impact on the stability of proteins compared with the hydrophobic amino acids present in a protein interior [40]. To conclude, the buriability scale proposed by [40] describes the residue–residue and residue–solvent interactions and was significantly and positively correlated with the hydrophobicity of a residue. Referring these findings to our results in the FR model, we observed that “Bur” of a C-terminal amino acid in a dipeptide sequence had an impact on log IC_50_ (positive correlation). No impact on the dipeptide log IC_50_ was observed for C-Hdr. Although, “Bur” is the parameter used to estimate protein stability [40], the lack of correlations between C-Hdr and C-Bur in the FR model could legitimate the application of buriability for the analysis of dipeptide structure–ACE-inhibition/bitterness. On the other hand, our results from the BR model indicated that N-Hdr of a residue (positive correlations) was in opposition to N-bur (negative correlations). Considering the fact that the buriability scale is useful for “residue-type” interactions (see above) [40], it can also be used to describe the interactions of short-chain peptides, such as ACE inhibitors with proteins (e.g., bitter taste receptors).

As it was said above, peptide bioactivity is determined by its amino acid composition [41]. When looking at both sets of ACE-inhibiting/bitter dipeptides found in Table 2, the dominant amino acids forming the peptide sequences were as follows: F, G, and R located at both the N- and C-end of a dipeptide chain. Some authors [42] have summarized the structural features affecting peptide bitterness. Apart from net hydrophobicity of a whole sequence, it was found that the presence of specific residues and their locations in a peptide chain were important as well. For example, peptides containing P within the sequence with the additional presence of R, V, and L (both N- and C-terminal) were indispensable for peptides to be bitter [42]. Such peptide sequences (i.e., containing P) could be also observed among the dipeptides possessing approximate observed versus predicted values of log IC_50_ with R_caf_. (see Table 2).

Although we could observe some similarities in the amino acid composition of bitter ACE inhibitors, which was reflected by the variables indicated in the FR and BR models, there were also some dipeptides with more diversified structures that did not match the above-described regularities. These results were reflected by relatively low correlations between the dipeptide ACE inhibition and bitterness obtained for the FR and BR models (see Table 3). According to other authors, although it is possible to find some correlations between ACE-inhibitory activity and the bitterness of the peptides, further direct comparison of these two properties is rather difficult due to one measure of activity applied. Usually, it is the measure describing ACE inhibition or bitterness [13]. 

To summarize the above discussion, our protocol, consisting of the application of curated databases and cheminformatic websites for the construction of data matrices, can be useful in chemometric analyses of the structure–function prediction of peptides with double bioactivity. Although we found FR to be statistically better than BR, considering that only the first one cannot be considered to be unequivocal. Both models indicated some common features, which influence the bitterness and ACE-inhibitory potential of dipeptides; however, looking at FR and BR in detail, they differed in the number of other descriptors important in the data interpretation. This is due to the fact that FR and BR are methods of variable selection, enabling the construction of models “eliminating” those whose impact on the dependent variable is negligible. However, FR and BR can be recommended for result interpretation. Such an approach allows for more consistent inferences and explanations of the relationship between the structure and ACE-inhibitory potential of bitter dipeptides. Our procedure and results obtained can be useful for structure–function analysis of peptides with other bioactivities and can be applicable when developing technologies for the production and/or elimination of peptides with desired/undesired properties.

## 4. Materials and Methods 

### 4.1. Dataset Construction

#### 4.1.1. Peptides 

The samples (i.e., objects and cases) taken to create a dataset were 28 dipeptide sequences acting simultaneously as ACE inhibitors and bitter tastants, i.e., FL(0.12), EY(0.43), PR(0.61), VY(0.85), VF(0.96), KP(1.34), KF(1.45), IL(1.74), RF(1.97), RP(2.26), AF(2.28), GY(2.32), GP(2.40), RR(2.43), FP(2.50), LF(2.54), GF(2.80), YP(2.86), IF(2.97), IG(3.08), GI(3.08), YG(3.18), GL(3.40), GR(3.51), FG(3.57), GV(3.66), GE(3.73), and LG(3.94). Their ACE-inhibiting measure was an IC_50_ value defined as the concentration [µM] of peptides corresponding to its half-maximal inhibition [13]. The abovementioned values assigned to each peptide were derived from the literature, which was cited in the “*References*” toolbar of the BIOPEP-UWM database of bioactive peptides [26,43]. Moreover, the specific value of IC_50_ can be found in the window called “EC_50_ [µM]”. It results from the universal graphical layout of the BIOPEP-UWM database of bioactive peptides containing sequences with over 40 bioactivities, which can be expressed as EC_50_, i.e., substance concentration giving half-maximal effect [44]. Thus, in the case of ACE-inhibiting peptides, their “EC_50_” found in the BIOPEP-UWM database toolbar should be understood as “inhibition” meaning IC_50_ [45].

Finally, the IC_50_ parameter of each dipeptide was transformed into a logarithm to enable multilinear regression analyses (see above in parens). 

#### 4.1.2. Variables

Values of log IC_50_ were dependent variables (see the chapter above). The independent variables were the descriptors (i.e., attributes and descriptors) associated with the physicochemical properties of N- and C-terminal amino acids forming ACE-inhibiting/bitter dipeptides. They were as follows: molecular weight (Molw), bulkiness (Bul) [46], polarity (Pol) [47], hydrophobicity (Hdr) [48], the number of carbon atoms (_at_C), the number of hydrogen atoms (_at_H), steric parameter (Str) [49], polarizability parameter (Polar) [50], size (Size) [51], and buriability (Bur) [40]. The abbreviations of variables are provided in parens. Moreover, all the abbreviations possessed an “N-” and “C-” prefix to associate an appropriate variable with the dipeptide N- and C-terminal amino acid residue, respectively. For example, a variable “C-_at_C” should be read as the number of carbon atoms in a C-terminal amino acid of a dipeptide sequence.

The abovementioned attributes were implemented from the following databases: ProtScale (Molw, Bul, Pol, and Hdr) [52,53], Biological Magnetic Resonance Data Bank (atC and atH) [54,55], and AAindex (Str, Polar, Size, and Bur) [56,57]. All the databases were accessed between April and June 2018.

### 4.2. Protocol

Multiple regression (MLR) for the ACE-inhibiting/bitter dipeptide dataset was made using STATISTICA^®^13.1 software (StatSoft, Cracow, Poland). The calculations were run by opening the “*Statistics*” menu and selecting the function “*Multiple Regression*”. After the selection of dependent and independent variables (see chapters above) and opening the toolbar “*Advanced*”, the following options were ticked: “*Advanced options (stepwise or ridge regression)*” and “*Extended precision computations*”. It enabled opening the window called “*Model Definition*”, selecting the function entitled “*Stepwise*” and then “*Forward stepwise”* and ticking “*At each step*” from the window called “*Display results*”. Choosing the option entitled “*At each step*” allowed for the observation of consecutive changes in the models during the procedure of particular model estimation (i.e., backward/forward stepwise regression models). An identical protocol was applied to make a backward stepwise regression by ticking the function called “*Backward stepwise”* instead of “*Forward stepwise”* in the “*Model Definition*” window. 

Statistical procedures run to check if the regression analyses were applied appropriately when considering the representativeness of the samples and variables were carried out according to the recommendations in [23]. For example, the statistical significance of the estimated regression equations was determined using the global Fisher–Snedecor test (F-test) [23] at α = 0.01 and verifying the null versus the alternative hypotheses (H_null_, H_alt_, respectively). H_null_ meant that the independent variables have no impact on the dependent variable. H_alt_ was in opposition to H_null_. Rejection of H_null_ indicated that at least 1 regression coefficient was non-zero. The statistical significance of particular predictors (i.e., regression coefficients; *b*) was analyzed by *t*-test at α = 0.01 [28].

## 5. Conclusions

The FR and BR models differed in R^2^, which suggests that the FR model is statistically better than the BR model in the structure–activity prediction of the ACE-inhibiting/bitter-tasting dipeptides. Both models showed some common properties, which determine the dual function of the dipeptides. The FR model revealed more descriptors important in data interpretation than did the BR model. The FR procedure led to finding the following statistically significant regularities, which determine the ACE-inhibitory/bitterness of dipeptides expressed by their log IC_50_: **C-_at_C(−)**, C-Bur(+), **N-Molw(+)**, N-_at_H(+), N-Pol(−). In turn, the BR model revealed the importance of additional variables, such as N-bul(−), N-Bur(−), and N-Hdr(+). The values in parens represent positive and negative correlations with log IC_50_, respectively, while the bold font shows statistically significant variables indicated in both the FR and BR models. The properties of the amino acids represented by individual variables affected the composition of the sequences analyzed. It was found that bitter ACE inhibitors preferred P, Y, F (C-end) and G, V, I, L (N-end). 

Based on the results obtained, FR and BR can be recommended as chemometric techniques for the prediction of relationships between the structure and activity of peptides, such as ACE inhibitors with bitter taste. Our protocol, including curated databases and programs containing chemometric data, can be also applicable for the structure–function analysis of peptides with other biological functions. 

## Figures and Tables

**Table 1:** Statistical summary of forward and backward regression (FR and BR, respectively) made for the angiotensin converting enzyme (ACE)-inhibiting/bitter dipeptide dataset (α = 0.01).

Statistical Data	Forward Regression (FR)	Backward Regression (BR)
F	(10,17) ^1^ 17.1	(5,22) ^1^ 14.1
R	0.95	0.87
R^2^	0.91	0.76
Adjusted R^2^	0.86	0.71
*p*	0.000001	0.000003
Standard estimation error	0.41	0.58
Statistically significant variables ^2^	C-_at_C, C-Bur, N-Molw, N-_at_H, N-Pol	N-Molw, N-Bul, N-Hdr, C-_at_C, N-Bur

^1^ levels of freedom (in parens); ^2^ C-_at_C - number of carbon atoms of C-terminal amino acid; C-Bur - buriability of C-terminal amino acid; N-Molw - molecular weight of N-terminal amino acid; N-_at_H - number of hydrogen atoms of N-terminal amino acid; N-Pol - polarity of N-terminal amino acid; N-Bul - bulkiness of N-terminal amino acid; N-Hdr - hydrophobicity of N-terminal amino acid; N-Bur - buriability of N-terminal amino acid.

**Table 2:** ACE-inhibiting and bitter-tasting dipeptides with approximate observed and predicted values of log IC_50_.

FR				BR			
Dipeptides	Log IC_50_		R_caf._^1^	Peptides	Log IC_50_		R_caf._
	Observed	Predicted			Observed	Predicted	
GR	3.51	3.43	0.01	GL	3.40	3.19	0.04
YP	2.86	2.83	0.05	LG	3.94	4.00	0.05
RR	2.43	2.56	0.13	RR	2.43	2.30	0.13
**FG** ^2^	3.57	3.55	0.17	**FG**	3.57	3.47	0.17
**GV**	3.66	3.62	0.22	**GV**	3.66	3.38	0.22
EY	0.43	0.57	0.25	IG	3.08	2.96	0.22
KP	1.34	1.38	0.33	YG	3.18	2.95	0.33
**PR**	0.61	0.69	0.33	**PR** ^2^	0.61	0.59	0.33
**VY**	0.85	0.84	0.33	**VY**	0.85	0.97	0.33
**VF**	0.96	1.10	0.33	**VF**	0.96	0.97	0.33
**RF**	1.97	1.98	0.4	**RF**	1.97	1.72	0.4
GE	3.73	3.58	0.67	GI	3.08	3.19	0.44
**LF**	2.54	2.59	0.77	**LF**	2.54	2.65	0.77
**GF**	2.80	2.85	0.83	**GF**	2.80	2.61	0.83
RP	2.26	2.17	1.25	**↑ Total: 14 (BR)**			
**↑ Total: 15 (FR)**				**Common dipeptides (9):** PR, VY, VF, RF, RR, LF, GF, FG, GV			

^1^ R_caf._—values adopted from [20]; ^2^ bold—peptides with identical sequences found in both models.

**Table 3:** Correlations between dipeptide ACE inhibition and bitterness obtained for the FR and BR models (α = 0.01).

Model	Log R_caf._ = *f* (Observed log IC_50_)		Log R_caf._ = *f* (Predicted log IC_50_)	
FR	R^2^ = 0.10	R = −0.32	R^2^ = 0.10	R = −0.22
BR	R^2^ = 0.05	R = −0.32	R^2^ = 0.05	R = −0.23
*p*	0.09		0.25	

## Abbreviations

ACE	Angiotensin converting enzyme
α-value	Significance level
BR	Backward regression
FR	Forward regression
F-test	Fisher–Snedecor test
IC_50_	Concentration of peptide corresponding to its half-maximal inhibitory activity [µM]
MLR	Multiple regression
*p*-value	Probability of a given statistical model
R	Correlation coefficient
R_caf._	Bitterness of peptide related to that of 1 mM caffeine solution
R^2^	Determination coefficient
SW-W	Normality Shapiro–Wilk’s test
*t*-test	Student’s *t* test
